# Efficient Production of Polyhydroxyalkanoate Through Halophilic Bacteria Utilizing Algal Biodiesel Waste Residue

**DOI:** 10.3389/fbioe.2021.624859

**Published:** 2021-09-16

**Authors:** Sonam Dubey, Sandhya Mishra

**Affiliations:** Applied Phycology and Biotechnology division, CSIR – Central Salt and Marine Chemicals Research Institute, Bhavnagar, India

**Keywords:** algal biodiesel waste residue, *Halomonas daqingensis*, *Halomonas ventosae*, Polyhydroxyalkanoates, optimization, characterization

## Abstract

The objective of the current work was to investigate the potential of halophilic bacterial isolates for efficient utilization of crude glycerol from algal biodiesel waste into polyhydroxyalkanoates (PHAs) a green plastic. Screening of the isolates was directly done in algal biodiesel waste residue containing solid agar plates supplemented with Nile red. Crude glycerol is a biodiesel waste whose bioconversion into value-added products provides an alternative for efficient management with dual benefit. For the scale-up studies of PHAs, *Halomonas spp.* especially *H. daqingensis* was observed as a potential candidate growing well in 3% Algal biodiesel waste residue (ABWR), 5% NaCl supplementation at 35°C within 48 h of incubation. Maximum Cell dry weight (CDW) of 0.362 ± 0.001 g and 0.236 ± 0.003 g PHA was obtained with *H. daqingensis* when grown in the fermentor with 0.5 vvm air flow rate and 200 rpm containing 3% ABWR supplemented with 5% NaCl at 35°C incubation temperature for 48 h. ABWR can serve as a sole substrate for PHA production at an industrial scale serving two approaches: getting rid of the biodiesel industrial waste containing high amount of glycerol besides using waste replacing commercial substrate thereby reducing the cost of the product.

## Introduction

Petroleum-based plastics are essential for widespread applicability in routine life. The major drawback of these synthetic plastics is its non-biodegradability and its toxic nature during incineration with the release of harmful gases into the environment. This has led to serious damage to the environment. In order to avoid these problems, the prerequisite is to produce biodegradable plastics and look for renewable resources as nutrient feed for its commercialization. Polyhydroxyalkanoates (PHAs) have gained interest amongst researchers due to its characteristic properties which are appropriate for its wide applicability.

Bioplastic production may increase from 2 Mt/y in 2017 to 2.4 Mt/y in 2022 as per the reports (Bioplastics Market Data, 2017 2017-2022). The present scenario affecting the cost of PHAs in the world market is the use of expensive substrates and downstream processing methodology (Kumar and Kim, 2018). Several substrates are reported in the literature which are exploited for PHA production *viz.* sugars, fatty acids, vinasses-molasses mixture, waste sludge, waste water, etc. (Frison et al., 2015; Huang et al., 2016; Koller et al., 2017; Liao et al., 2018; García et al., 2019). Amongst the cheap substrates explored so far, crude glycerol is the potential feedstock for PHA production also enhancing the sustainability of biodiesel industry (Shrivastav et al., 2010; Ghosh et al., 2015a,b). Biodiesel production may reach 41 Mm^3^ by 2022 (Monteiro et al., 2018). The major byproduct of biodiesel industry is glycerol which needs to be dealt with for waste management systems. It is thus of great importance that crude glycerol is used as feed material for the production of polyhydroxyalkanoates exploiting wild strain of halophilic bacteria. Value added utilization of crude glycerol obtained during biodiesel production is essential as it is important for various industrial processes and products. Glycerol is therefore exploited as carbon substrate for the production of PHAs by several researchers (Freches and Lemos, 2017; Ntaikou et al., 2018; Kumar and Kim, 2018). The ever increasing price for synthetic nutrient media has led us use crude substrates e.g., glycerol generated as a waste stream during biodiesel production (Shrivastav et al., 2010; Bera et al., 2015). This could make the production of PHA from renewable resources more competitive with common plastics with almost zero effluent for biodiesel industries thus, lowering down the overall processing cost. Many fermentable and non-fermentable ingredients present in the crude glycerol generated through various methods could hinder the overall bio-process (Bhattacharya et al., 2016; Gahlawat and Soni, 2017; Fauzi et al., 2019). Thus, a suitable strain with proper growth optimized conditions utilizing the biodiesel co-product for fermentation is essential. Several researchers have worked upon reducing the input of synthetic substrates for PHA productivity. In few studies, they have investigated the glycerol as a suitable substrate for bacterial growth (Garlapati et al., 2016). Crude glycerol contains mainly fatty acids, methanol and salts which if accumulated in higher concentration during the growth of bacteria may hinder the fermentation process Rahman et al. (2015) (Bhattacharya et al., 2016) utilized wastewater microalgae-based media for the production of PHA from recombinant *E.coli*. Many halophilic bacteria are reported to accumulate PHAs (Kucera et al., 2018; Kumar and Kim, 2018; Hong et al., 2019). Halophilic bacteria have gradually emerged as an interesting candidate of research due to its ease of cultivation with less chances of contamination owing to its extremophilic nature. Several methodologies were adapted by researchers for the exploitation of halophilic bacteria for PHA production (Cervantes-Uc et al., 2014).

Extensive use of synthetic plastics as packaging materials has found applicability in several areas. Their cheap, light weight and material properties are appealing behind their widespread demand. The problem in disposing these plastics raises harsh environmental issues. Biodegradable plastics like polyhydroxyalkanoates (PHAs) with similar material properties and affordability would suitably replace such hazardous synthetic plastics. Biodegradation of PHA was reported by Madbouly et al. (2014) to be increased with the addition of distiller’s dried grains with solubles. Torri et al. (2017) reported the conversion of PHA-containing sludge into alkenes and CO_2_ through hydrothermal treatment. Scaling up the PHA production requires lowering down the production cost (Choi and Lee, 1999). Factors affecting the cost of the product are the fermentation substrate required, fermentation time, raw materials, type of bacteria downstream processing, efficient recovery process, purity, etc. with thermal and mechanical properties of polymer (Rodriguez-Perez et al., 2018). On the basis of its physicochemical and mechanical properties its applications could be decided. Few researchers explored the use of sugars from crops as bacterial feed, but it is not feasible during scaling up of the process as these crops are also consumed by humans as primary food. One of the important factors affecting the process is exploring potential wild bacterial strain capable of growing in open saline system. This reduces the chance of contamination and also the harvesting time which in turn saves energy during scale up of process.

Herein, we evaluated the growth and polymer productivity in halophilic bacteria. Taking into consideration the bottleneck concerning the commercial media utilization for PHA production, algal biodiesel waste residue (ABWR) was used as the sole nutrient source. To the best of our knowledge this is the first report on PHB production by *Halomonas ventosae* and *Halomonas daqingensis* isolated from the Experimental Salt Farm (ESF) (21°47′26.4″N 72°07′19.2″E) utilizing algal biodiesel waste residue as the sole production media without addition of any external nutrient sources.

## Materials and Methods

### Direct Screening

Direct screening of PHA accumulating halophilic bacteria on Nile red dye agar plates helped us avoid extensive use of costly commercial media for screening purpose. To check the capability of bacteria to utilize glycerol as the carbon source for PHA production, the samples (0.1 mL) were spread on the minimal salt medium plates with varying NaCl concentration ranging from 3-25%. Samples collected were salt, soil and water samples from different salt pans (with varying salinity) Kumbharwada, Bhavnagar, Gujarat (21°47′26.4″N 72°07′19.2″E). The minimal salt medium (MSM) consists of Ammonium sulfate, 0.5 g/l; magnesium sulfate, 0.4 g/l; disodium hydrogen phosphate, 9.65 g/l; potassium dihydrogen phosphate, 2.65. Micronutrient solution composition: ferrous sulfate, 2.78 g/l; manganese chloride, 1.98 g/l; cobalt sulfate, 2.81 g/l; copper chloride, 0.17 g/l; zinc sulfate, 0.29 g/l containing synthetic glycerol and supplemented with a solution of 0.25 mg Nile red in DMSO. The plates were incubated for 48–120 h at 30°C to check the bacterial growth which was further checked for fluorescence under UV light in a Gel doc (Biorad, United States) (Supplementary Figure 2). These PHA positive isolates were picked up and restreaked onto respective ZMA plates supplemented with NaCl and maintained further for secondary screening.

The isolates showing better fluorescence were selected for shake flask batch experiments. The media used as inoculum was Zobell marine broth supplemented with NaCl ranging from 5–25%. The experiments were setup in 500 ml Erlenmeyer flask with 100 ml production media containing algal biodiesel waste residue. The experiment was setup till 96 h of incubation and maximum biomass and polymer productivity was monitored every 24 h interval. The cells were harvested by centrifugation at 10,000 rpm for 10 min. The cell pellets were further dried at 60°C for 24 h. The cell dry weight was noted gravimetrically and further PHA was extracted through sodium hypochlorite (4%) treatment from the dried biomass. The cell pellets and sodium hypochlorite was vortexed thoroughly until a homogeneous mixture is obtained followed by centrifugation. The pellets were washed with water and further with methanol and allowed to dry. The obtained polymer was dissolved in chloroform and films were prepared and measured gravimetrically.

### Bacterial Strains

Two potential bacterial isolates *Halomonas ventosae* (GenBank KY953158) and *Halomonas daqingensis* (GenBank KY962964) were selected on algal biodiesel waste residue containing agar plates (Supplementary Figure 3). These isolates were further maintained on Zobell Marine agar (ZMA) supplemented with 3% NaCl containing slants at 4°C. The cultures were maintained in glycerol stocks at −80°C.

### The Seed Culture

The seed culture was raised by growing the bacterial culture on Zobell marine broth/dry sea mix (DSM) (Ghosh et al., 2015b; Mishra et al., 2014; Dhangdhariya et al., 2015) supplemented with 3% (w/v) sodium chloride, pH 7.6 ± 0.2 at 30°C for 18 h and absorbance of the culture broth was noted at 600 nm in UV-visible spectrophotometer (Varian Cary Bio 50).

### Optimization of Process Parameters for Maximum Polymer Productivity

Factors affecting bacterial growth and polymer accumulation in the promising bacterial isolates capable of utilizing algal biodiesel waste residue consisting of crude glycerol; additional sodium chloride concentrations; agitation/static; temperature; size of inoculum and pH. All the experiments were carried out in triplicates.

#### Optimization of Growth Parameters Using Algal Biodiesel Waste Residue

In this study, varying concentrations of algal biodiesel waste residues (ABWR) containing crude glycerol on growth and PHA accumulation by isolates *Halomonas daqingensis* and *Halomonas ventosae* were studied. The crude glycerol was prepared by the process mentioned in detail in supporting information (Supplementary Figure 1). Both the isolates were inoculated in medium containing algal biodiesel waste residue with varying concentrations *viz*. 1, 2, 3, 4, and 5%. Both biomass and polymer productivity were recorded.

#### Effect of NaCl Concentration Supplemented in Production Media

Time course study of NaCl concentration on growth and PHA accumulation by *H. daqingensis* and *H. ventosae* was checked by inoculating the overnight grown culture (Optical density = 1) in production media containing varying concentration of NaCl *viz.* 5, 10, 15, 20, and 25%.

#### Effect of pH in Production Media

The pH plays a crucial role for bacterial metabolism. Thus, we tried to optimize this condition for its better growth and polymer accumulation. The production medium prepared was adjusted to varying pH values ranging from 5.0, 6.0, 7.0, 8.0, and 9.0 with 0.1 N HCL or 5 M NaOH solutions prior to sterilization.

#### Effect of Temperature

Optimization of temperature conditions were carried out at various temperatures *viz*. 30°C, 32°C, 35°C, 37°C, 40°C, and 50°C. Overnight grown culture was inoculated in the production media and incubated further at various temperatures. Biomass and PHA productivity were recorded.

#### Effect of Agitation and Static Condition

The role of agitation and static condition on bacterial growth and PHA production was checked by inoculating the culture in one set of production media kept in static condition while the other set was incubated at 120 rpm shaking condition in an Orbitek shaker at 35°C. The production media consisted of 3% and 4% ABWR with pH 7 for *H. daqingensis* and *H. ventosae* respectively.

#### Effect of Inoculum Size

Inoculum size plays a critical role in the PHA productivity experiments. *H. daqingensis* and *H. ventosae* were inoculated with varying concentrations ranging from 5% v/v, 10% v/v, 15% v/v and 20% v/v in 3% and 4% ABWR medium respectively.

### Fermentor Studies

*Halomonas daqingensis* was further selected for PHA productivity study at fermentor level due to its rapid growth and PHA accumulation which saves energy during scale-up process. The overnight grown culture of *H. daqingensis* (15%) was inoculated in 5 L fermentor for batch fermentation process with a working volume of 2 L using the optimized medium.

The media components were 3% ABWR and 5% NaCl and made up to 1700 ml with distilled water and pH was set to pH 7 using 5M NaOH. The media was further sterilized and 300 mL of overnight grown culture was seeded aseptically. The temperature was maintained at 35°C. No antifoaming agents were incorporated into the production media. The air inflow rate and agitation speed were initially adjusted to 0.5 vvm and 200 rpm, respectively, during the fermentation process. Samples (ca. 50 ml) were withdrawn after every 24 h interval and were checked for growth and PHA productivity.

### Statistical Optimization

Statistical optimization was carried out through Design Expert software 8.0 to ascertain the interactive effects of different variables through response surface methodology (RSM). Based on the optimum results, central composite design was prepared and the most significant variables ABWR, NaCl and pH were checked.

The three distinct variables used were ABWR, NaCl and pH denoted as A, B and C respectively employed to optimize the fermentation conditions and thereby to obtain maximum PHB yield. An experimental design of 20 runs (experiments) was formulated using the Design Expert software. All experiments were conducted in 500 ml Erlenmeyer flasks with 100 mL total working volume prepared according to the design and inoculated with 15 ml of seed culture. The production flasks were kept on a rotary shaker maintained at 35°C and 120 rpm. Response calculated was PHA (g) at the end of 48 h for *H. daqingensis.* The contour surface plots were created to understand the interaction of different factors, and the graphs were used to evaluate the optimized components and conditions of the medium, which influences the responses. The point prediction is a unique feature of this software which was used to confirm the obtained optimum value.

### Extraction of Polyhydroxyalkanoate From Bacterial Biomass

The culture was harvested through centrifugation which was dried at 60°C overnight and treated with 4% sodium hypochlorite solution. The biomass was thoroughly vortexed until a uniform mixture is formed followed by centrifugation at 10000 rpm for 10 min. The obtained pellets were washed with water and methanol consecutively. The obtained pellets were dissolved in chloroform and the solvent was allowed to evaporate and PHA film was obtained.

### Chemical Analysis of Extracted Polymer

#### Thermal Gravimetric Analysis (TGA)

The thermal properties of the extracted polymer were checked using thermogravimetric analysis using TG-DTA system in TG 209 F1 instrument. The sample was analyzed over a temperature range of 500°C at a heating rate of 10°C min^–1^ under nitrogen atmosphere.

#### Fourier Transform Infrared Spectroscopy (FT-IR)

The extracted polymer was mixed with KBr spectra was noted in 4000 – 400 cm^–1^ range with PerkinElmer spectrum GX FTIR spectrometer in by mixing the extracted polymer with.

#### Nuclear Magnetic Resonance (NMR)

The extracted polymer was dissolved in deuterated chloroform for ^1^H NMR analysis at 500 MHz and compared with standard PHB (Sigma Aldrich).

#### GPC Analysis

The Column used was 2 PL Gel Mixed D (300 mm × 7 mm) with Guard column in series. Mobile Phase CHCl3 stabilized with 1% Ethanol and polystyrene was used as calibration standard.

## Results and Discussion

Exploitation of waste substrates like crude glycerol from biodiesel for PHA production helps in getting rid of waste management problems and thereby decreasing environmental concerns too (Ibrahim and Steinbüchel, 2009; Zhu et al., 2013). Many researchers have worked on the utilization of synthetic substrates which will add to the cost of the product. A wide range of raw substrates are explored for its potential use for bacterial growth (Raza et al., 2018; Volova et al., 2019). The experiments were performed in triplicate and Tukey’s test was applied for data interpretation in the data analyzed by applying the Tukey’s test using the MStat-C software.

### Optimization Studies

#### Optimization of Algal Biodiesel Waste Residue Concentration

**FIGURE 1:**
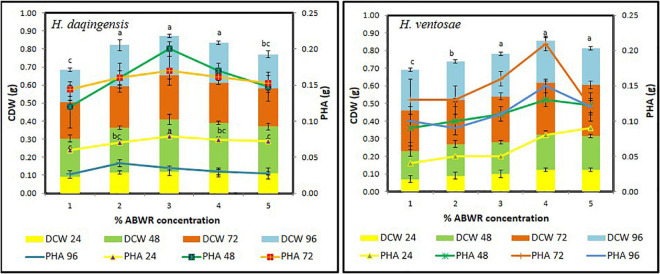
Growth kinetics and PHA accumulation of *Halomonas daqingensis* and *Halomonas ventosae* respectively in varying concentration of ABWR.

Increasing the algal biodiesel waste residue concentration decreases the total organic carbon utilization, which shows that, both isolates do not utilize the organic carbon present in the medium completely. The optimum concentration for growth and PHA production is 30 g/l and 40 g/l respectively for both the isolates *Halomonas daqingensis* (Figure 1) and *Halomonas ventosae* (Figure 1) respectively.

The cell dry weight (CDW) obtained was significantly high with 0.29 ± 0.03 g/100 mL at 30 g/L ABWR concentration at 48 h incubation for *Halomonas daqingensis* and 0.29 ± 0.01 g/100 mL for *Halomonas ventosae* at 40 g/L ABWR concentration after 72 h of incubation. PHA content of 0.20 ± 0.02 g/100 mL and 0.21 ± 0.01 g/100 mL was obtained in *Halomonas daqingensis* and *Halomonas ventosae* respectively. Increase in the concentration above 3% ABWR in *H. daqingensis* decreased both the bacterial growth and PHA accumulation which clearly indicates that high concentration of ABWR may be detrimental for bacterial growth whereas, concentrations above 4% ABWR does not affect much of *H. ventosae* growth. A similar growth trend was observed at different time intervals with varying concentration which depicts the optimum time of harvesting to be 48 h and 72 h for *H. daqingensis* and *H. ventosae* respectively. Whole genome sequenced *Halomonas hydrothermalis* is reported to accumulate co-polymer when fed with crude levulinic acid and *Jatropha* biodiesel wastes (Bera et al., 2015; Bharadwaj et al., 2015).

#### Effect of Sodium Chloride on Cell Growth and PHA Production

**FIGURE 2:**
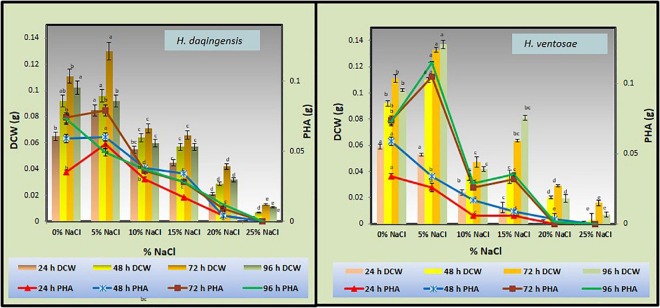
Effect of NaCl on biomass and PHA productivity in both *Halomonas daqingensis* (D) and *Halomonas ventosae* (V).

Sodium chloride is one of the constituent of crude glycerol derived from biodiesel during alkaline catalyzed transesterification of the algal oil. Thus, the effect of additionally supplemented sodium chloride on growth and PHA accumulation in the isolates was estimated by inoculating the cultures in medium containing different concentrations of sodium chloride *viz*. 5, 10, 15, 20, and 25% and no additional NaCl (denoted as 0%) in algal biodiesel waste residue. It was observed from the graph that NaCl content affects the rate of bacterial growth affecting its lag phase. In case of *Halomonas daqingensis* with no additional NaCl highest CDW was observed at 48 h of incubation while increasing the NaCl concentration to 5% increased the lag phase shifting the optimum growth at 72 h of incubation (Figure 2).

Highest PHA productivity was observed at 72 h of incubation with additional 5% NaCl concentration. In *Halomonas ventosae* the growth increased at 96 h of incubation with 5% NaCl concentration depicting the large lag phase (Figure 2).

Moderately halophilic bacteria grow in the presence of 3 to 15% salt concentration while, extremely halophilic bacteria require more than 15% of salt concentration. Several ions such as NaCl is essential for the growth and stability of these micro-organisms both growth and metabolism of these bacteria (Gibbons, 1969).

#### Effect of pH on the Biomass and PHA Accumulation

**FIGURE 3:**
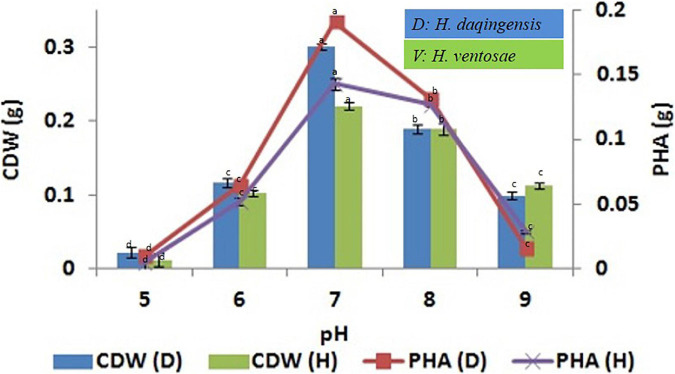
Effect of pH on biomass and PHA productivity in both *Halomonas daqingensis* and *Halomonas ventosae.*

The influence of initial pH on biomass and PHA productivity by *H. daqingensis* and *H. ventosae* was checked using 3% ABWR and 4% ABWR production media respectively. The optimum pH for PHA productivity for both the isolates was pH 7 with 0.191 ± 0.002 g and 0.143 ± 0.005 g PHA in *H. daqingensis* and *H. ventosae* respectively. Increasing the pH above 7 decreases PHA accumulation in both the isolates as observed from the Figure 3.

#### Effect of Temperature on the Biomass and PHA Productivity

**FIGURE 4:**
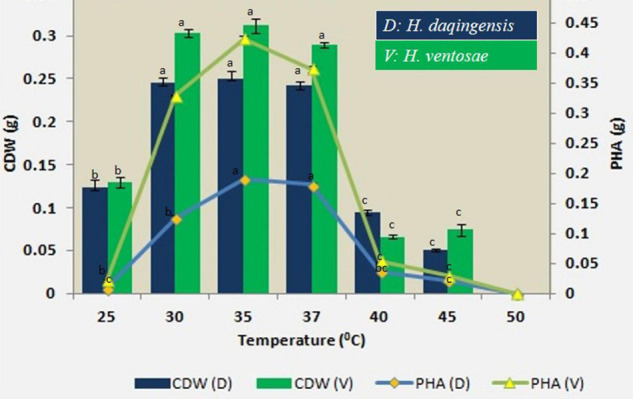
Effect of temperature on biomass and PHA productivity in both *Halomonas daqingensis* and *Halomonas ventosae*.

The role of temperature on biomass and PHA productivity was checked in *H. daqingensis* and *H. ventosae* at different incubation temperatures *viz.* 25°C, 30°C, 35°C, 40°C, 45°C and 50°C. The maximum PHA accumulation was obtained at 35 °C temperature for both the isolates as indicated in Figure 4.

#### Effect of Static and Agitation on the Growth and Polymer Accumulation

The effect of agitation and static condition on growth and PHA accumulation was estimated by incubating one set of the inoculated medium in agitated condition in a shaker at 120 rpm and incubating the other set of inoculated medium in static condition. The medium constituent was 3% and 4% algal biodiesel waste residue for *H. daqingensis* and *H. ventosae* respectively incubated at 35°C. *H. daqingensis* showed 0.12g of CDW with 0.06g PHA under static condition while 0.33g CDW and 0.24g PHA under agitation condition. In case of *H. ventosae* 0.11 g CDW with 0.05 g PHA accumulation under static condition while 0.26 g CDW and 0.18 g PHA accumulation was found to be under agitation condition. As observed from these results, shaking condition is an important parameter for growth and PHA accumulation due to maximum oxygen transfer rate in the culture medium enhancing the bacterial metabolism for efficient accumulation of PHAs.

#### Effect of Inoculum Size on the Biomass and PHA Accumulation

**FIGURE 5:**
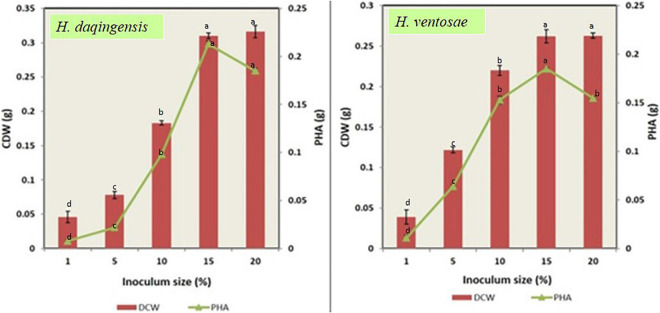
Role of inoculum size on biomass and PHA productivity in both *Halomonas daqingensis* and *Halomonas ventosae*.

Inoculum size is also one of the important factor responsible in the optimization process. Both the isolates *Halomonas daqingensis* and *Halomonas ventosae* were inoculated into the production medium with varying inoculum size of 1% v/v, 5% v/v, 10% v/v, 15% v/v, 20% v/v in 30 g/L and 40 g/l of algal biodiesel waste. Figure 5 indicates bacterial growth along with polymer accumulation.

An increase in CDW and polymer content was observed in both the isolates from the graph till 15%v/v inoculum. Thus, maximum growth with 0.310 ± 0.04 g and polymer accumulation with 0.213 ± 0.03 g was recorded at 15% v/v concentration in *H. daqingensis* whereas 0.262 ± 0.08 g biomass and 0.185 ± 0.01 PHA productivity in *H. ventosae.* Increasing the inoculum size to 20% v/v decreased both CDW and polymer content.

### Scaling Up of the Optimized Process in Bioreactor

Fermentor studies was carried out under optimized conditions utilizing *H. daqingensis.* In the present experiment, the inexpensive ABWR was used as production media with additional 5% NaCl. Samples were withdrawn every 24 h time interval to check possible bacterial growth and PHA production. The centrifuged pellets were collected for PHA. PHA extraction and supernatant was filter sterilized (0.45 μ) and stored at 4°C until used for total organic carbon analysis. The results obtained indicated potential utilization of cheap substrates as nutrient source for bacterial growth and PHA production at industrial scale. The un-optimized conditions resulted in 0.362 ± 0.001 g of dried bacterial biomass and 0.236 ± 0.003 g of PHA within 48 h of incubation.

### Statistical Optimization

The study was conducted using Design Expert software version 8. Three different variables: ABWR (1-8%), NaCl (0-10%) and pH (6-8) was considered for the experiment. The experiment was set up at 100 mL scale in 500 mL Erlenmeyer flasks containing 85 mL of production medium and 15 mL of inoculum, 35°C incubation temperature, 48 h production age, 120 rpm agitation in an Orbitek shaker. The above mentioned medium was considered for seed culture medium. To estimate the effect of different factors on PHA as response surface, central composite design was used. Based on the results obtained by optimizing different factors through one factor at a time approach, the ranges of factors were selected. The experimental data derived from the design were analyzed by multi regressions through the least square method to fit the below second order polynomial equation. A quadratic model was obtained in this study.

Y is the measured response variable, coefficients βo, βi, βij, and βii are constant which represent either synergistic or antagonistic effects for different combinations and regression coefficient of the model and xi and xj represents the independent variables in coded values. The predictive power was verified using Fischer (F) through analysis of variance (ANOVA) determining the level of significance. Also, *p*-values (Prob. > F) less than 0.05 is preferable. (Tables 1, 2)

**TABLE 1:** Role of agitation and static condition on bacterial growth and polymer accumulation.

**Incubation**	***H. daqingensis***		***H. ventosae***	
	**CDW (g)**	**PHA (g)**	**CDW (g)**	**PHA (g)**
**Static**	0.12	0.06	0.11	0.05
**Agitation**	0.33	0.24	0.26	0.18

**TABLE 2:** ANOVA for response surface quadratic model.

**Analysis of variance table**					
	**Sum of**		**Mean**	**F**	**p-value**
**Source**	**Squares**	**df**	**Square**	**Value**	**Prob > F**
Model	0.048	9	5.32	21.38	< 0.0001
A-ABWR	6.28	1	6.28	25.24	0.0005
B-NaCl	0.016	1	0.016	65	< 0.0001
C-pH	1.25	1	1.256	5.04	0.0485
AB	4.51	1	4.51	18.12	0.0017
AC	4.05	1	4.05	0.16	0.6952
BC	3.28	1	3.28	13.18	0.0046
A^2^	0.012	1	0.012	48.66	< 0.0001
B^2^	1.03	1	1.03	4.17	0.0684
C^2^	5.45	1	5.45	21.89	0.0009
Residual	2.49	10	2.49		
Lack of Fit	2.20	5	4.41	7.79	0.0209
Pure Error	2.83	5	5.66		
Cor Total	0.050	19			

**FIGURE 6:**
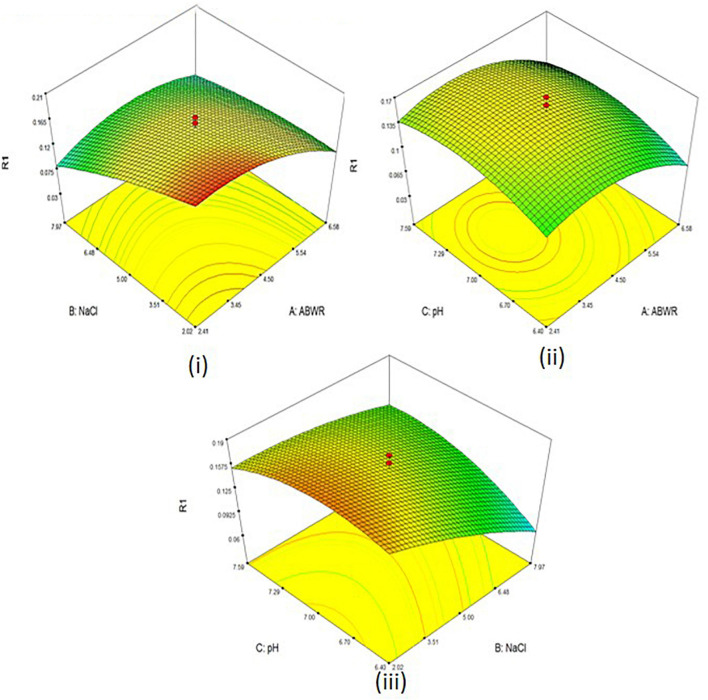
3D surface plots of PHA productivity with (i) ABWR and NaCl (ii) ABWR and pH (iii) NaCl and pH.

A three- factor, five level central composite design (CCD) was employed to check the effects of different combinations on polymer accumulation (Figure 6 **and** Table 3).

**TABLE 3:** CCD for PHA optimization ABWR (1-8%); NaCl (0-10%); pH (6-8).

**Std**	**ABWR (%)**	**NaCl (%)**	**pH**	**PHA (g)**
1	2.41	2.02	6.40	0.21
2	6.58	2.02	6.40	0.09
3	2.41	7.97	6.40	0.024
4	6.58	7.97	6.40	0.06
5	2.41	2.02	7.59	0.15
6	6.58	2.02	7.59	0.1
7	2.41	7.97	7.59	0.106
8	6.58	7.97	7.59	0.09
9	0.99	5	7	0.115
10	8	5	7	0.03
11	4.5	0	7	0.19
12	4.5	10	7	0.071
13	4.5	5	5.99	0.079
14	4.5	5	8	0.12
15	4.5	5	7	0.16
16	4.5	5	7	0.15
17	4.5	5	7	0.17
18	4.5	5	7	0.16
19	4.5	5	7	0.16
20	4.5	5	7	0.15

The concentrations of the different factors were set as per our preliminary experiments conducted at the same level. The experiment consisted of 20 runs. The Model F-value of 21.38 suggests the model is significant. There is only a 0.01% chance that a “Model F-value” this large might be due to noise. Final equation for PHA derived in terms of coded factors:

0.16 + −0.021 ^∗^ A + −0.034 ^∗^ B + 9.589E - 0.03 ^∗^ C + 0.024 ^∗^ A^∗^ B +

2.250E + −003 ^∗^ A^∗^ C + 0020 ^∗^ B^∗^ C + −0.029 ^∗^ A^2^ + −8.489E-003 ^∗^ B^2^ + −0.019 ^∗^ C^2^

The maximum productivity with 0.21 g was observed at 2.41% ABWR; 2.02% NaCl and 6.40 pH.

### Extraction and Characterization of the Extracted Polymer

The supernatant after centrifugation of the bacterial biomass was filter sterilized (0.45μ) and stored at 4 °C until use. This filter sterilized supernatant was analyzed for organic carbon content which was 14.45% at 48 h of incubation. Extraction was carried out using conventional technique utilizing 4% sodium hypochlorite. Dubey et al., 2017 reported the extraction of PHA utilizing green solvents. Also, the dissolution of PHA was reported in green solvent diethanol ammonium acetate with maximum solubility of 3% w/v at 70°C (Sequeira et al., 2020). Herein, the extracted polymer showed good thermal and mechanical properties compared to standard PHB from Sigma Aldrich. The detailed description of the characterization experiments are mentioned in Supporting information file (Supplementary Figures 2, 3) as Table 4 above indicates the comparative PHA productivity in different bacterial strains compared with our present study.

**TABLE 4:** Comparative analysis of PHA production.

**Sr No.**	**Strain**	**Feedstock**	**Working volume**	**Crude glycerol (%)**	**Temperature**	**PHA productivity**	**References**
1.	Mixed culture	Crude glycerol; volatile fatty acids;1,3-propanedio	1.7 L	1	37 ^0^C	-	Burniol-Figols et al., 2018
2.	*P. putida* KT2440	Crude glycerol	1.5 L	3%	30 ^0^C	47%	Poblete-Castro et al., 2014
3.	*Novosphingobium sp.*	Crude glycerol	-	2%	-	45%	Teeka et al., 2012
4.	*Bacillus thuringiensis*	Crude glycerol; Nutrient broth	125mL	2%	37 ^0^C	74.8%	Kumar et al., 2015
4.	*Pannonibacter phragmitetus ERC8*	Crude glycerol	200 mL	0.8%	30 ^0^C	17.41%	Ray et al., 2016
5.	*Bacillus sp. ISTVK1*	Pure glycerol	-	5%	30 ^0^C	85.19%	Morya et al., 2018
6.	*Paracoccus sp. strain LL1*	Glycerol	150 mL	2%	30 ^0^C	39.3%	Kumar and Kim, 2018
7.	*Halomonas daqingensis*	Crude glycerol	100 mL	3%	35 ^0^C	68.96%	This study
8.	*Halomonas ventosae*	Crude glycerol	100 mL	4%	35^0^C	72.41%	This study

#### Comparative TGA Profiles of PHA Extracted at Different Time Intervals With Standard PHB (Sigma Aldrich)

The thermal degradation temperature of extracted PHA from *H. daqingensis* and *H. ventosae* were observed to be 290°C and 296°C which is higher than detected for commercial PHB (Sigma Aldrich). The increase in degradation temperature for observed biomass can be due to trace amount of impurities which was further checked through elemental C,H,N,S analysis. Extracted PHA sample was checked for impurities left after extraction process through Elemental analysis. The extracted sample contains 55.95% and 56.56% carbon which is similar to that of standard PHB obtained from Sigma Aldrich.

#### Fourier Transform Infrared Spectroscopy (FT-IR)

The FT-IR spectra of PHB (Sigma Aldrich) displayed characteristic peaks, ν-OH str. at 3436 cm-1, ν-CH2 and ν-CH3 str. at 2880 to 2977 cm-1, ν-C = O str.at 1724 cm-1 and ν-C-O str. at 1000-1300 cm-1. The recovered polymer (both from *H. daqingensis* and *H. ventosae*) exhibited all specific peaks, consistent to that detected for PHB (Sigma Aldrich). Hence, it was definite that the recovered polymer is PHB

#### Nuclear Magnetic Resonance (NMR) Analysis

The ^1^H-NMR spectra of PHB indicated doublet at δ = 1.28 and 1.27 ppm due to methyl group, doublet of quadruplet at δ = 2.58 and 2.49 ppm, ascribed to the methylene group, multiplet at δ = 5.25 ppm is characteristic due to methine group.

#### Dynamic Mechanical Analysis (DMA)

The robustness of the PHA film reduced with the rise in temperature as depicted in the graph. The E′ became constant at around 125°C which designates the glass transition temperature though, the glass transition recorded through DSC was around 175°C which can be due to the sensitivity of instruments.

#### Gas Permeation Chromatography (GPC) Analysis

The extracted polymer has molecular weight of 309 KDa extracted from *H. daqingensis* with 1.82 PDI.

### Comparative Production Analysis of PHA Utilizing Glycerol

Polyhydroxyalkanoates accumulation in halophilic bacteria in the presence of high salts is beneficial not requiring sterile conditions thus preventing growth of wild bacterial strains.

### Novelty of the Current Study and Its Significant Contribution

The novelty of the current study is utilization of ABWR as sole substrate for PHA production in both the halophilic bacterial strains i.e., *H. daqingensis* and *H. ventosae.* These two strains are capable of accumulating PHA within 48–72 h of incubation which decreases the overall production cost.

## Conclusion

The grail of the current work was to reconnoiter and formulate the process for the exploitation of algal biofuel wastes containing crude glycerol for the production of polyhydroxyalkanoate that can replace commercial substrates. The priority in our current study for selecting salt pans as sample collection site was its extreme environment nurturing several bacteria adapted in such harsh environment. This wonder niche is a focal point for potential microbes *viz. Halomonas daqingensis* and *Halomonas ventosae*, as an efficient PHA producer with maximum PHA productivity in presence of 3% and 4% ABWR concentration respectively at 35°C temperature.

## Data Availability Statement

The original contributions presented in the study are included in the article/Supplementary Material, further inquiries can be directed to the corresponding author/s.

## Author Contributions

SD performed the conceptualization, methodology, conducting experiments and writing. SM did the conceptualization, editing and reviewing. Both authors contributed to the article and approved the submitted version.

## Conflict of Interest

The authors declare that the research was conducted in the absence of any commercial or financial relationships that could be construed as a potential conflict of interest.

## Publisher’s Note

All claims expressed in this article are solely those of the authors and do not necessarily represent those of their affiliated organizations, or those of the publisher, the editors and the reviewers. Any product that may be evaluated in this article, or claim that may be made by its manufacturer, is not guaranteed or endorsed by the publisher.

## References

[B1] Bera A. Dubey S. Bhayani K. Mondal D. Mishra S. Ghosh P. K. (2015). Microbial synthesis of polyhydroxyalkanoate using seaweed-derived crude levulinic acid as co-nutrient. *Int. J. Biol. Macromol* 72 487–494. 10.1016/j.ijbiomac.2014.08.037 25193103

[B2] Bharadwaj S. V. Shrivastav A. Dubey S. Ghosh T. Paliwal C. Maurya R. (2015). Draft genome sequence of *Halomonas hydrothermalis* MTCC 5445, isolated from the west coast of India. *Genome Announc.* 3:e01419-14. 10.1128/genomeA.01419-14 25593258 PMC4299900

[B3] Bhattacharya S. Dubey S. Singh P. Shrivastava A. Mishra S. (2016). Biodegradable polymeric substances produced by a marine bacterium from a surplus stream of the biodiesel industry. *Bioengineering* 3:34. 10.3390/bioengineering3040034 28952596 PMC5597277

[B4] Bioplastics Market Data, (2017). *Global Production Capacities of Bioplastics 2017–2022. Report European Bioplastics.* Available online at: https://www.european-bioplastics.org/market.

[B5] Burniol-Figols A. Varrone C. Daugaard A. E. Le S. B. Skiadas I. V. Gavala H. N. (2018). Polyhydroxyalkanoates (PHA) production from fermented crude glycerol: study on the conversion of 1, 3-propanediol to PHA in mixed microbial consortia. *Water Res.* 128 255–266. 29107910 10.1016/j.watres.2017.10.046

[B6] Cervantes−Uc J. M. Catzin J. Vargas I. Herrera−Kao W. Moguel F. Ramirez E. (2014). Biosynthesis and characterization of polyhydroxyalkanoates produced by an extreme halophilic bacterium, *Halomonas nitroreducens*, isolated from hypersaline ponds. *J. Appl. Microbiol.* 117 1056–1065. 25048168 10.1111/jam.12605

[B7] Choi J. Lee S. Y. (1999). Factors affecting the economics of polyhydroxyalkanoate production by bacterial fermentation. *Appl. Microbiol. Biotechnol.* 51 13–21. 10.1007/s002530051357

[B8] Dhangdhariya J. H. Dubey S. Trivedi H. B. Pancha I. Bhatt J. K. Dave B. P. (2015). Polyhydroxyalkanoate from marine *Bacillus megaterium* using CSMCRI’s Dry Sea Mix as a novel growth medium. *Int. J. Biol. Macromol.* 76 254–261. 10.1016/j.ijbiomac.2015.02.009 25697675

[B9] Dubey S. Bharmoria P. Gehlot P. S. Agrawal V. Kumar A. Mishra S. (2017). 1-Ethyl-3-methylimidazolium diethylphosphate based extraction of bioplastic “Polyhydroxyalkanoates” from bacteria: green and sustainable approach. *ACS Sustain. Chem. Eng.* 6 766–773. 10.1021/acssuschemeng.7b03096

[B10] Fauzi A. H. M. Chua A. S. M. Yoon L. W. Nittami T. Yeoh H. K. (2019). Enrichment of PHA-accumulators for sustainable PHA production from crude glycerol. *Process. Saf. Environ. Prot.* 122 200–208. 10.1016/j.psep.2018.12.002

[B11] Freches A. Lemos P. C. (2017). Microbial selection strategies for polyhydroxyalkanoates production from crude glycerol: effect of OLR and cycle length. *New Biotechnol.* 39 22–28. 10.1016/j.nbt.2017.05.011 28587886

[B12] Frison N. Katsou E. Malamis S. Oehmen A. Fatone F. (2015). Development of a novel process integrating the treatment of sludge reject water and the production of polyhydroxyalkanoates (PHAs). *Environ. Sci. Technol.* 49 10877–10885. 10.1021/acs.est.5b01776 26270064

[B13] Gahlawat G. Soni S. K. (2017). Valorization of waste glycerol for the production of poly (3-hydroxybutyrate) and poly (3-hydroxybutyrate-co-3-hydroxyvalerate) copolymer by *Cupriavidus necator* and extraction in a sustainable manner. *Bioresour. Technol.* 243 492–501. 10.1016/j.biortech.2017.06.139 28692918

[B14] García C. Alcaraz W. Acosta-Cárdenas A. Ochoa S. (2019). Application of process system engineering tools to the fed-batch production of poly (3-hydroxybutyrate-co-3-hydroxyvalerate) from a vinasses molasses mixture. *Bioproc. Biosystems. Eng.* 42 1023–1037. 10.1007/s00449-019-02102-z 30874887

[B15] Garlapati V. K. Shankar U. Budhiraja A. (2016). Bioconversion technologies of crude glycerol to value added industrial products. *Biotechnol. Rep.* 9 9–14. 10.1016/j.btre.2015.11.002 28352587 PMC5360980

[B16] Ghosh P. K. Mishra S. C. P. Gandhi M. R. Upadhyay S. C. Paul P. Anand P. (2015a). *U.S. Patent No. 8,956,836.* Washington, DC: U.S. Patent and Trademark Office.

[B17] Ghosh P. K. Upadhyay S. C. Mishra S. Mohandas V. P. Srivastava D. V. Shahi V. K. (2015b). *U.S. Patent 20,150,004,673.* Bhavnagar: CSIR-CSMCRI.

[B18] Gibbons N. E. (1969). “Chapter VIII isolation, Growth and requirements of halophilic bacteria,” in *Methods in Microbiology*, Vol. 3 eds Norris J. R. Ribbons D. W. (Cambridge, MA: Elsevier Ltd.), 169–183. 10.1016/S0580-9517(08)70507-5

[B19] Hong J. W. Song H. S. Moon Y. M. Hong Y. G. Bhatia S. K. Jung H. R. (2019). Polyhydroxybutyrate production in halophilic marine bacteria *Vibrio proteolyticus* isolated from the Korean peninsula. *Bioproc. Biosystems. Eng.* 42 603–610. 10.1007/s00449-018-02066-6 30617415

[B20] Huang L. Liu C. Liu Y. Jia X. (2016). The composition analysis and preliminary cultivation optimization of a PHA-producing microbial consortium with xylose as a sole carbon source. *Waste Manag.* 52 77–85. 10.1016/j.wasman.2016.03.020 27021696

[B21] Ibrahim M. H. Steinbüchel A. (2009). Poly (3-hydroxybutyrate) production from glycerol by *Zobellella denitrificans* MW1 via high-cell-density fed-batch fermentation and simplified solvent extraction. *Appl. Environ. Microbiol.* 75 6222–6231. 10.1128/AEM.01162-09 19666728 PMC2753068

[B22] Koller M. Maršálek L. de Sousa Dias M. M. Braunegg G. (2017). Producing microbial polyhydroxyalkanoate (PHA) biopolyesters in a sustainable manner. *New Biotechnol.* 37 24–38. 10.1016/j.nbt.2016.05.001 27184617

[B23] Kucera D. Pernicová I. Kovalcik A. Koller M. Mullerova L. Sedlacek P. (2018). Characterization of the promising poly (3-hydroxybutyrate) producing halophilic bacterium *Halomonas halophila*. *Bioresour. Technol.* 256 552–556. 10.1016/j.biortech.2018.02.062 29478784

[B24] Kumar P. Kim B. S. (2018). Valorization of polyhydroxyalkanoates production process by co-synthesis of value-added products. *Bioresourc. Technol.* 269 544–556. 10.1016/j.biortech.2018.08.120 30201320

[B25] Kumar P. Ray S. Patel S. K. Lee J. K. Kalia V. C. (2015). Bioconversion of crude glycerol to polyhydroxyalkanoate by *Bacillus thuringiensis* under non-limiting nitrogen conditions. *Int. J. Biol. Macromol.* 78 9–16. 25840150 10.1016/j.ijbiomac.2015.03.046

[B26] Liao Q. Guo L. Ran Y. Gao M. She Z. Zhao Y. (2018). Optimization of polyhydroxyalkanoates (PHA) synthesis with heat pretreated waste sludge. *Waste Manag.* 82 15–25. 10.1016/j.wasman.2018.10.019 30509577

[B27] Madbouly S. A. Schrader J. A. Srinivasan G. Liu K. McCabe K. G. Grewell D. (2014). Biodegradation behavior of bacterial-based polyhydroxyalkanoate (PHA) and DDGS composites. *Green Chem.* 16 1911–1920.

[B28] Mishra S. C. P. Ghosh P. K. Gandhi M. R. Bhattacharya S. Maiti S. Upadhyay S. C. (2014). *US20140099684A1.* Bhavnagar: CSIR-CSMCRI.

[B29] Monteiro M. R. Kugelmeier C. L. Pinheiro R. S. Batalha M. O. da Silva César A. (2018). Glycerol from biodiesel production: technological paths for sustainability. *Renew. Sustain. Energy Rev.* 88 109–122. 10.1016/j.rser.2018.02.019

[B30] Morya R. Kumar M. Thakur I. S. (2018). Utilization of glycerol by *Bacillus* sp. ISTVK1 for production and characterization of polyhydroxyvalerate. *Bioresourc. Technol. Rep.* 2 1–6.

[B31] Ntaikou I. Koumelis I. Tsitsilianis C. Parthenios J. Lyberatos G. (2018). Comparison of yields and properties of microbial polyhydroxyalkanoates generated from waste glycerol based substrates. *Int. J. Biol. Macromol.* 112 273–283. 10.1016/j.ijbiomac.2018.01.175 29391227

[B32] Poblete-Castro I. Binger D. Oehlert R. Rohde M. (2014). Comparison of mcl-Poly (3-hydroxyalkanoates) synthesis by different *Pseudomonas* putida strains from crude glycerol: citrate accumulates at high titer under PHA-producing conditions. *BMC Biotechnol.* 14:962. 10.1186/s12896-014-0110-z 25532606 PMC4299480

[B33] Rahman A. Putman R. J. Inan K. Sal F. A. Sathish A. Smith T. (2015). Polyhydroxybutyrate production using a wastewater microalgae based media. *Algal res.* 8 95–98.

[B34] Ray S. Prajapati V. Patel K. Trivedi U. (2016). Optimization and characterization of PHA from isolate *Pannonibacter phragmitetus* ERC8 using glycerol waste. *Int. J. Biol. Macromol.* 86 741–749. 26851207 10.1016/j.ijbiomac.2016.02.002

[B35] Raza Z. A. Abid S. Banat I. M. (2018). Polyhydroxyalkanoates: characteristics, production, recent developments and applications. *Int. Biodeterior. Biodegrad.* 126 45–56. 10.1016/j.ibiod.2017.10.001

[B36] Rodriguez-Perez S. Serrano A. Pantión A. A. Alonso-Fariñas B. (2018). Challenges of scaling-up PHA production from waste streams. A review. *J. Environ. Manag.* 205 215–230. 10.1016/j.jenvman.2017.09.083 28987985

[B37] Sequeira R. A. Dubey S. Pereira M. M. Maity T. K. Singh S. Mishra S. (2020). Neoteric solvent systems as a sustainable media for dissolution and film preparation of poly-[(R)-3-hydroxybutyrate]. *ACS Sustain. Chem. Eng.* 8 12005–12013. 10.1021/acssuschemeng.0c02684

[B38] Shrivastav A. Mishra S. K. Shethia B. Pancha I. Jain D. Mishra S. (2010). Isolation of promising bacterial strains from soil and marine environment for polyhydroxyalkanoates (PHAs) production utilizing *Jatropha* biodiesel byproduct. *Int. J. Biol. Macromol.* 47 283–287. 10.1016/j.ijbiomac.2010.04.007 20417229

[B39] Teeka J. Imai T. Reungsang A. Cheng X. Yuliani E. Thiantanankul J. (2012). Characterization of polyhydroxyalkanoates (PHAs) biosynthesis by isolated *Novosphingobium* sp. THA_AIK7 using crude glycerol. *J. Ind. Microbiol. Biot.* 395 749–758. 10.1007/s10295-012-1084-2 22286712

[B40] Torri C. Weme T. D. O. Samorì C. Kiwan A. Brilman A. D. W. (2017). Renewable alkenes from the hydrothermal treatment of polyhydroxyalkanoates-containing sludge. *Environ. Sci. Technol.* 51 12683–12691. 10.1021/acs.est.7b03927 28991443

[B41] Volova T. Demidenko A. Kiselev E. Baranovskiy S. Shishatskaya E. Zhila N. (2019). Polyhydroxyalkanoate synthesis based on glycerol and implementation of the process under conditions of pilot production. *Appl. Microbiol. Biotechnol.* 103 225–237. 10.1007/s00253-018-9460-0 30367183

[B42] Zhu C. Chiu S. Nakas J. P. Nomura C. T. (2013). Bioplastics from waste glycerol derived from biodiesel industry. *J. Appl. Polym. Sci.* 130 1–13. 10.1002/app.39157

