# Supplementary material for: Efficient Production of Polyhydroxyalkanoate Through Halophilic Bacteria Utilizing Algal Biodiesel Waste Residue
Source: Front Bioeng Biotechnol. 2021 Sep 16;9:624859. doi: 10.3389/fbioe.2021.624859 (PMC8481892; doi:10.3389/fbioe.2021.624859)
Supplement: Supplementary file 1 [file Data_Sheet_1.docx]

**Efficient production of polyhydroxyalkanoate through halophilic bacteria utilizing algal biodiesel waste residue**

Sonam Dubey, Sandhya Mishra*

**Supporting information**

- 1. **Purification of raw glycerol**

The composition of raw algal biodiesel waste is 49% glycerol; element (mg/l) Aluminium 0.231, Calcium 0.962, Cobalt 0.001, Chromium 0.015, Copper 0.027, Iron 0.257, Potassium 63.26, Magnesium 0.633, Manganese 0.011, Molybdenum 0.020, Sodium 6.044, Nickel 0.011, Zinc 0.288; methanol 12%; free fatty acids 44%. This raw ABWR was heated at 60 °C temperature in a rotary evaporator for methanol removal from raw ABWR. The resultant material was further treated with concentrated sulphuric acid creating acidic environment for soap separation when heated at 50 °C. This material was left overnight in a separating funnel. Next day, the lower layer was collected and neutralized with 5 N NaOH. The resultant material with 70% glycerol was analysed for glycerol content using commercially available free glycerol reagent (Sigma, Aldrich).

**Figure S1: Purification of raw glycerol.**


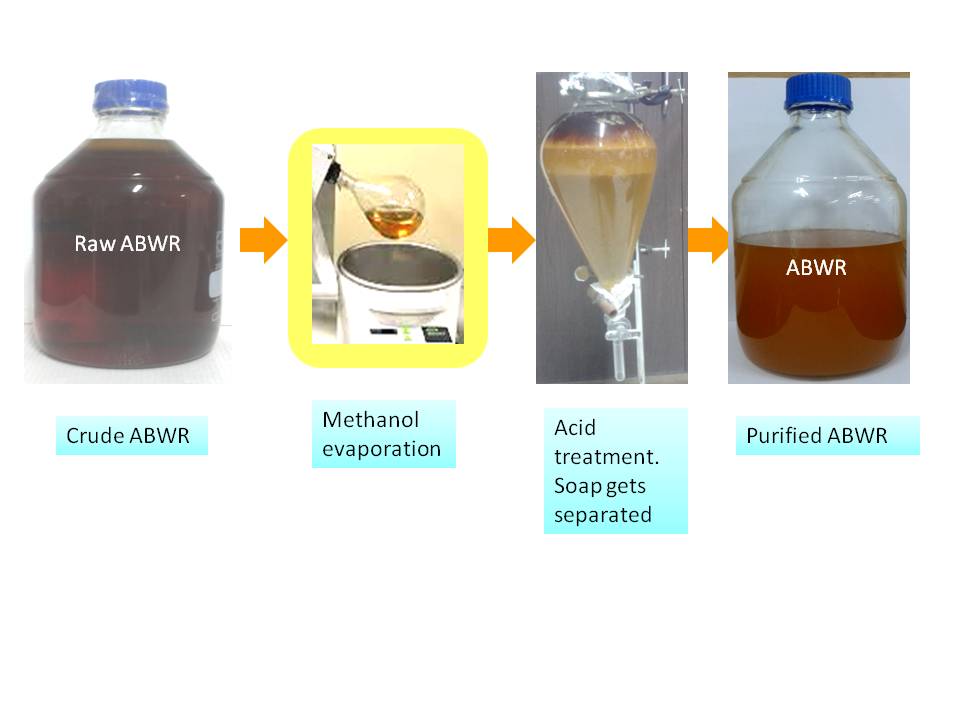


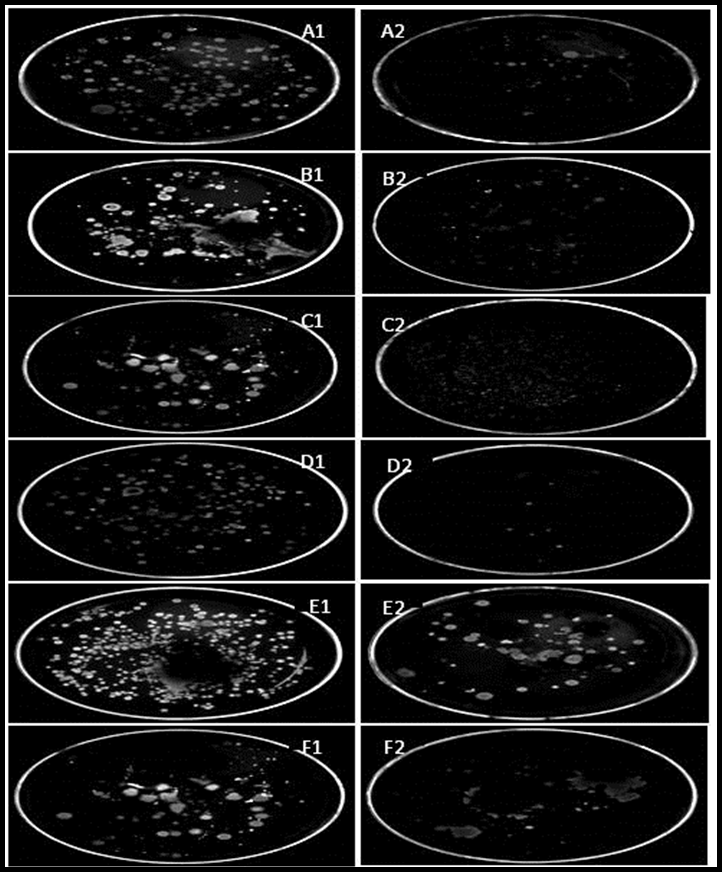


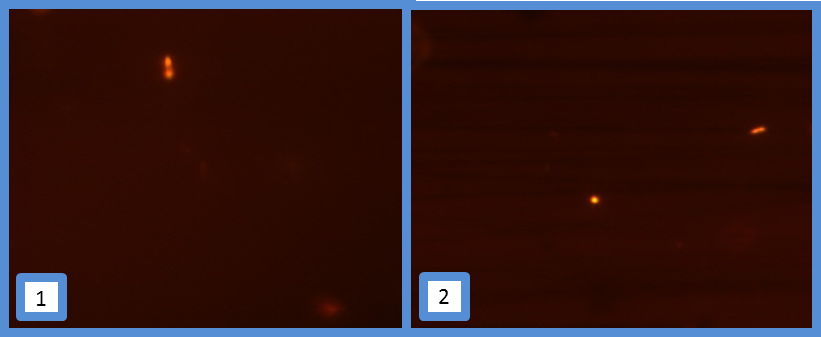


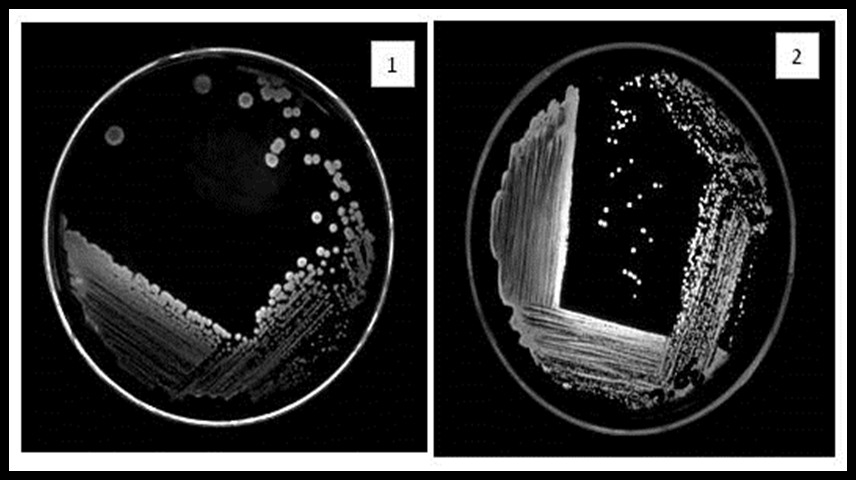


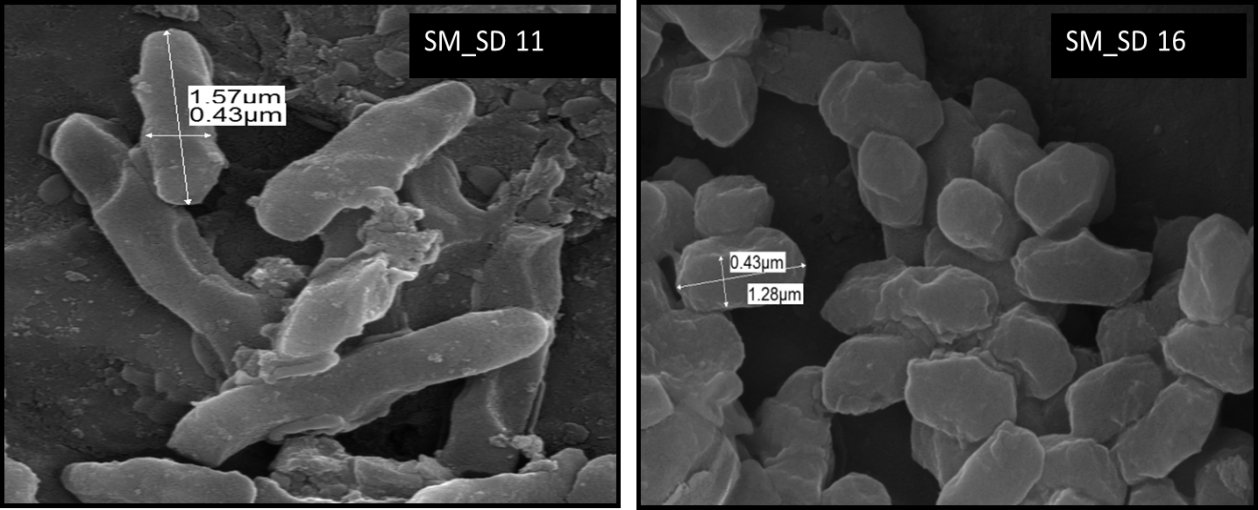


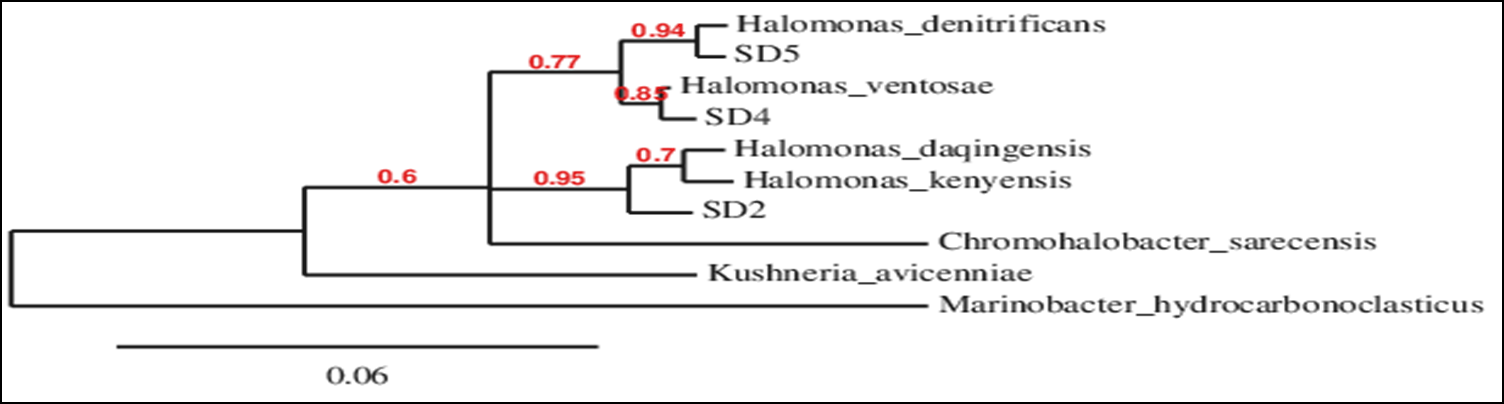


- 1. **Characterization**

**1.2.1.Thermal gravimetric analysis**

The thermal properties of the extracted polymer were checked using thermogravimetric analysis using TG-DTA system in TG 209 F1 instrument. The sample was analysed over a temperature range of 500^°^C at a heating rate of 10 ^°^C min^−1^ under nitrogen atmosphere.

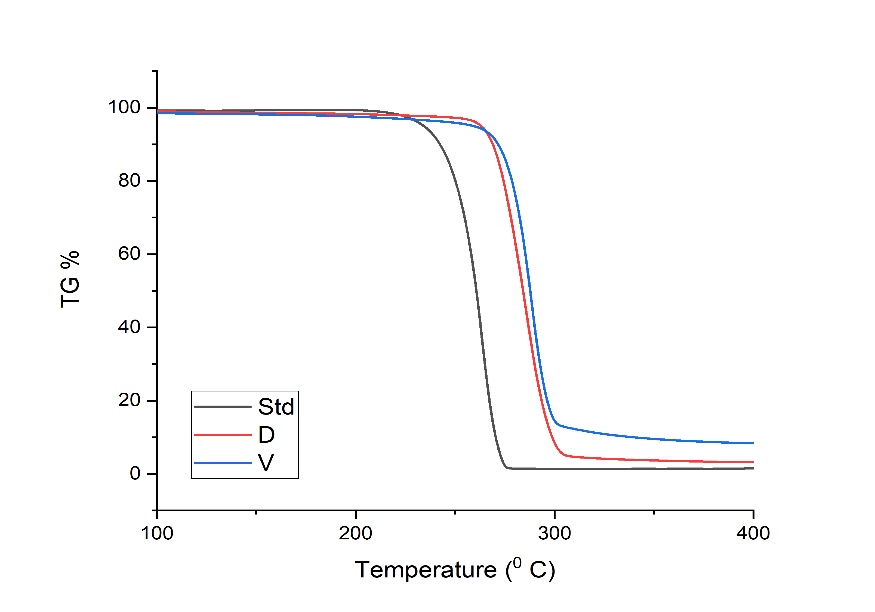


Figure S2: TGA analysis of std: Standard PHB from Sigma Aldrich; polymer extracted from D: *Halomonas daqingensis*; V: *Halomonas ventosae*

**1.2.2. Nuclear Magnetic Resonance (NMR):** The extracted polymer was dissolved in deuterated chloroform for ^1^H NMR analysis at 500 MHz and compared with standard PHB (Sigma Aldrich).

**

**

Figure S3: NMR analysis of polymer extracted from *Halomonas daqingensis* and standard PHB from Sigma Aldrich

**1.2.3. GPC analysis**

The Column used was 2 PL Gel Mixed D (300 mm x 7 mm) with Guard column in series. Mobile Phase CHCl_3_ stabilised with 1% Ethanol and polystyrene was used as calibration standard.

The extracted polymer has molecular weight of 309 KDa extracted from *H. daqingensis* with 1.82 polydispersity index.
